# Inhibition of CK2 mitigates Alzheimer’s tau pathology by preventing NR2B synaptic mislocalization

**DOI:** 10.1186/s40478-022-01331-w

**Published:** 2022-03-04

**Authors:** Courtney A. Marshall, Jennifer D. McBride, Lakshmi Changolkar, Dawn M. Riddle, John Q. Trojanowski, Virginia M.-Y. Lee

**Affiliations:** Department of Pathology and Laboratory Medicine, Institute on Aging and Center for Neurodegenerative Disease Research, University of Pennsylvania Perelman School of Medicine, Philadelphia, PA 19104 USA

**Keywords:** Casein Kinase 2, Alzheimer’s disease, NR2B, Memantine, Tauopathy

## Abstract

Alzheimer’s disease (AD) is a neurodegenerative disorder that exhibits pathological changes in both tau and synaptic function. AD patients display increases in hyperphosphorylated tau and synaptic activity. Previous studies have individually identified the role of NR2B subunit-containing NMDA receptors in AD related synaptic dysfunction and aggregated tau without reconciling the conflicting differences and implications of NR2B expression. Inhibition of extrasynaptically located NR2B mitigates tau pathology in AD models, whereas the inhibition of synaptic NR2B replicates tau-associated hyperactivity. This suggests that a simultaneous increase in extrasynaptic NR2B and decrease in synaptic NR2B may be responsible for tau pathology and synaptic dysfunction, respectively. The synaptic location of NR2B is regulated by casein kinase 2 (CK2), which is highly expressed in AD patients. Here, we used patient brains diagnosed with AD, corticobasal degeneration, progressive supranuclear palsy or Pick’s disease to characterize CK2 expression across these diverse tauopathies. Human derived material was also utilized in conjunction with cultured hippocampal neurons in order to investigate AD-induced changes in NR2B location. We further assessed the therapeutic effect of CK2 inhibition on NR2B synaptic distribution and tau pathology. We found that aberrant expression of CK2, and synaptically translocated NR2B, is unique to AD patients compared to other tauopathies. Increased CK2 was also observed in AD-tau treated neurons in addition to the mislocalization of NR2B receptors. Tau burden was alleviated in vitro by correcting synaptic:extrasynaptic NR2B function. Restoring NR2B physiological expression patterns with CK2 inhibition and inhibiting the function of excessive extrasynaptic NR2B with Memantine both mitigated tau accumulation in vitro. However, the combined pharmacological treatment promoted the aggregation of tau. Our data suggests that the synaptic:extrasynaptic balance of NR2B function regulates AD-tau pathogenesis, and that the inhibition of CK2, and concomitant prevention of NR2B mislocalization, may be a useful therapeutic tool for AD patients.

**Supplementary Information:**

The online version contains supplementary material available at 10.1186/s40478-022-01331-w.

## Introduction

Casein Kinase 2 (CK2) is a constitutively active serine/threonine-specific protein kinase. CK2 is a tetrameric complex comprised of catalytic (α, α’) and regulatory, non-catalytic subunits (β) [39]. The catalytic activity of CK2 regulates various processes ranging from neuronal development and differentiation to DNA damage and repair [29, 45]. This highly conserved kinase also plays a role in cell death and survival in addition to metabolism [37, 62]. The multitude of regulatory functions designates CK2 as a life-essential protein, as knock-out studies have demonstrated that loss of CK2 is embryonic lethal [6]. CK2 is ubiquitously expressed in both nervous and peripheral tissues [25, 59]. In vivo rodent studies have demonstrated that CK2 is particularly expressed in the hippocampus, one of the primary regions initially afflicted in Alzheimer’s Disease (AD) [3, 25]. AD is a neurodegenerative proteinopathy defined by the misfolding and aggregation of the microtubule-associated protein tau, neuron loss and gliosis. The hyperphosphorylation of tau proteins co-assemble to form paired helical filaments (PHF) that yield the formation of neurofibrillary tangles (NFTs). Previous studies have demonstrated aberrant expression of CK2 in AD patients. Increased levels of CK2 were observed in both neurons and astrocytes within the hippocampus of AD patients compared to age-matched controls [65]. Further, immunohistochemical assays have revealed CK2 staining localized to both NFTs and tau hyperphosphorylated at serine 396/404 (PHF1) in AD patients [36, 47]. Significant increases in CK2 are also observed in primary neurons transfected with human tau, and C57/BL6 mice develop cognitive deficits indicative of AD after the overexpression of hippocampal CK2 [90].

One of CK2’s phosphorylation targets is the NR2B subunit containing NMDA receptor. In the adult nervous system, NR2B subunits are preferentially localized to the extrasynaptic space with minimal surface expression at the synaptic cleft [57]. NR2B subunits comprise postsynaptic glutamate receptors that regulate synaptic activity. Specifically, NR2B regulates long term depression (LTD), with a reduction in synaptic NR2B expression or the activation of extrasynaptic NR2B subunits resulting in an increase in neuronal firing [44, 48]. NR2B-mediated LTD specifically induces the phosphorylation of tau at serine 396/404 (PHF1) as opposed to other epitopes such as serine 202/threonine 305 [64]. Hyperactivity induced by a global decrease in NR2B function reproduces the early hippocampal synaptic deficits seen in both AD patients and transgenic mouse models of AD [56, 61]. Moreover, increases in extrasynaptic NR2B are associated with hyperphosphorylated tau in vitro and in vivo and pharmacological inhibition of NR2B down regulates tau pathology [1, 73, 75, 86]. This suggests that changes in NR2B synaptic location simultaneously facilitate different pathological aspects of AD, with decreases in synaptic NR2B resulting in synaptic dysfunction and increases in extrasynaptic NR2B yielding an aggregation of pathological tau. Phosphorylation of CK2 at tyrosine 255 (CK2_tyr255_) increases the kinase’s catalytic activity thereby inducing the internalization and extrasynaptic translocation of NR2B [17]. Enzymatically active CK2 phosphorylates NR2B at serine 1480 (NR2B_ser1480_) thereby detaching the NMDA subunit from its PSD95 synaptic anchor and facilitating its concomitant internalization or reinsertion to the extrasynaptic space [11, 66, 67]. This sequence of events results in both a decrease in synaptic NR2B and an increase in extrasynaptic NR2B, without disrupting the physiological number of receptors.

Previous in vitro studies have not yet determined if AD patient derived pathological tau induces an increase in CK2 or the mislocalization of NR2B. In addition, changes in phosphorylated or total CK2 and NR2B expression have not been investigated in relation to phosphorylated tau in the context of other tauopathies, such as corticobasal degeneration (CBD), progressive supranuclear palsy (PSP), and Pick’s disease (Pick’s). Therefore, in this study we characterized hippocampal expression of CK2 and NR2B in the postmortem brains of well characterized patients who died as a result of AD, CBD, PSP or Pick’s disease. After identifying significant changes solely in AD patients, we further examined the relationship between CK2_tyr255_ and NR2B_ser1480_, and PHF1 in postmortem AD brains. We observed a positive correlation between the two phosphorylated proteins and PHF1 specific to the dentate gyrus (DG) and cornu amonis (CA) region known as CA3, notwithstanding an equal expression of phosphorylated proteins throughout the hippocampus. We used AD patient derived PHFs (ADPHFs) and mouse primary hippocampal neurons to recapitulate AD-mediated changes in CK2 and NR2B in vitro. We hypothesized that the CK2 inhibitor 4,5,6,7-tetrabromobenzotriazole (TBB) would ameliorate the aggregation of pathological tau as well as NR2B mislocalization associated with AD. This in vitro approach was also used to elucidated the mechanistic contributions of NR2B location and function in tau aggregation.

## Materials and methods

### Human tissue and purification of human derived pathological tau

Human brain samples from 26 AD, 13 CBD, 19 PSP, and 10 Pick’s cases were used in this study in addition to 5 cases of individuals characterized with a Braak score of ≤ 2 for controls (Additional file 4:Table 1). All cases met their respective diagnostic criteria and were selected from the CNDR brain bank [77, 78]. Postmortem AD brains exhibiting abundant frontal AD-like tau pathology were selected for extraction to generate purified ADPHFs. Isolation and purification of pathological AD-tau was performed as previously described [22, 23, 26, 34]. Briefly, grey matter was extracted for brain homogenates in nine volumes of high-salt buffer (10 mm Tris with 0.8 m NaCl, pH 7.4) with 0.1% Sarkosyl and 10% sucrose. Samples were spun at 10,000 × g for 10 min at 4 ºC. Pellets were re-extracted twice using the same buffer conditions. Additional Sarkosyl was added to the pooled supernatants to reach 1%. Samples were spun by rotatation for 1 h at room temperature then centrifuged at 100,000 × g for 60 min at 4 ºC. The resulting 1% Sarkosyl-insoluble pellets containing pathological AD-tau were resuspended in PBS. The resuspended pellets were further purified by brief sonication with a handheld probe (Qsonica XL-2000), followed by centrifugation at 150,000 × g for 30 min at 4 ºC. The final purified supernatants contained insoluble pathological AD-tau and were used in subsequent experiments.

### Primary neuronal culture and transduction of AD-tau

Primary hippocampal neurons were prepared from outbred CD1 E16-E18 mouse embryos. Neurons were digested from tissue with papain (Worthington Biochemical Corporation). The dissociated neurons were resuspended in neural basal medium (Gibco, 21,103) with 2% B27, 1 × Glutamax, and 1 × penicillin/streptomycin (Gibco). Cells were plated with a density 100,000 cells per coverslip in 24-well plates. Coverslips were coated with PDL (poly-D-lysine, 0.1 mg/ml, Sigma-Aldrich) and borate buffer (0.05 M boric acid, pH 8.5). On DIV0, 10% FBS was added to the medium, which was replaced with fresh medium 1 day in vitro (DIV). AD-tau was diluted into PBS and sonicated then added on top of cells. Neurons were treated with 1 µg/well of AD-tau at DIV 7 and 2 µg/well at DIV 14. Cells treated with AD-tau at DIV 7 received CK2 inhibitory post-treatment with the IC_50_ dose of TBB (0.1 µM) at DIV 14. An equal dose of TBB was added 30 min prior to the addition of AD-tau on DIV 14 as pre-treatment. The IC_50_ dose of Memantine (0.1 µM) was used as a post-treatment at DIV 14. Immunocytochemistry was conducted at DIV 21 as previously described [30, 87].

### Histology and immunofluorescence staining

Neuropathologically diagnosed patients were selected for use in these studies. Available clinical information determined patient inclusion in MMSE, age of onset, and disease duration analyses. Results displayed in graphs represent individual immunohistochemical experiments for the representative protein of interest and available hippocampal patient material, representing one section frame/patient/experiment. Individual patients and n numbers vary across graphs accordingly. Results from each experiment were analyzed independently to account for batch variability. Human hippocampal tissues were fixed in 10% normal buffered formalin (10% NBF) and embedded in paraffin. Fixed tissue was cut into 6 µm thick sections and mounted on glass slides. Brain tissue was deparaffinized and rehydrated prior to immunostaining. Primary hippocampal neurons were fixed with cold methanol at −20 ºC for 20 min. All samples incubated with primary antibodies overnight at 4 ºC and with Alexa Fluor-conjugated secondary antibodies at room temperature for 2 h (Additional file 5: Table 2). Sudan black (0.3% solution) was used to quench autoflorescence. Cell nuclei within all samples were labeled with Fluoromount-G containing DAPI (Southern Biotech) or hematoxylin (Sigma-Aldrich). Human sections were scanned with a Lamina Multilabel Slide scanner (PerkinElmer). The image analysis platform HALO (Indica Labs) was used for quantification of both human and neuronal immunofluorescent staining, as previously described [30]. Briefly, immunofluorescent quantification was determined by the area occupied by the antibody signal multiplied by the intensity and normalized to the area signal x intensity of DAPI or hematoxylin. Images from photomicrographs were captured with either a light (Eclipse Ni, Nikon) or confocal microscope (Leica TCS SP8 STED 3X).

### Statistical analyses

Analysis of human and neuronal data involved a one-way ANOVA followed by a Tukey post hoc test. Correlational analysis was executed with Pearson’s correlation. All analyses were completed by Prism software (GraphPad Software, Inc). Statistical significance was defined by p ≤ 0.05. All data are presented as mean ± standard deviation.

## Results

### AD patient brains uniquely display increased hippocampal CK2

**Fig. 1:**
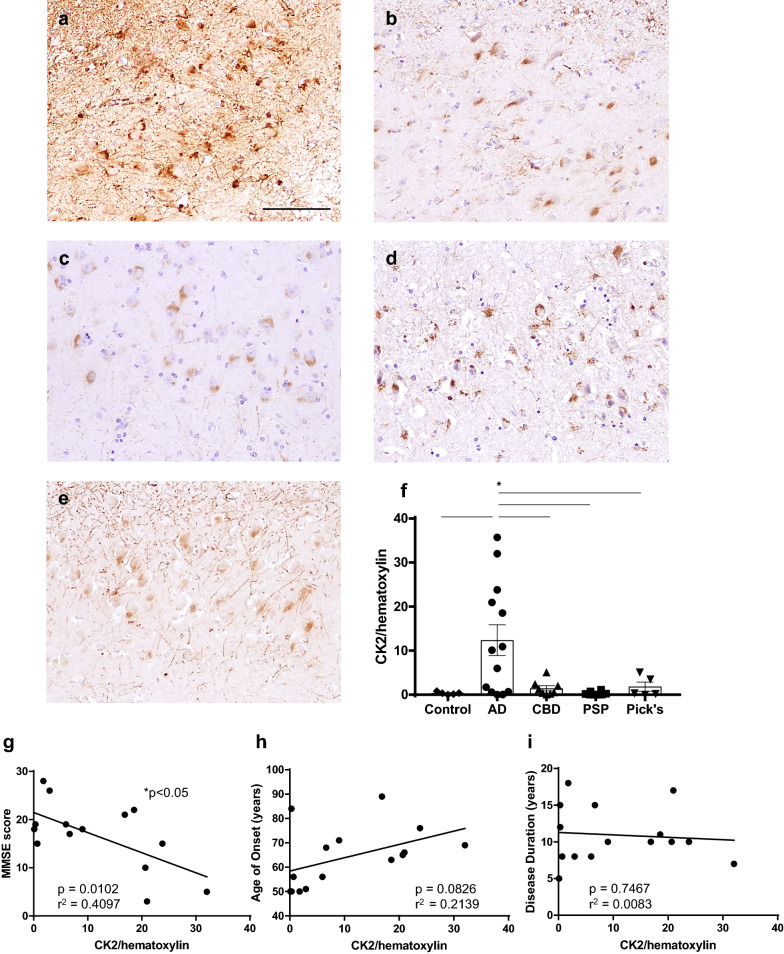
AD patient brains uniquely display increased hippocampal CK2. Hippocampal tissue samples from tauopathy patients and age-matched controls were stained with CK2 and counterstained with hematoxylin. **a** AD, **b** CBD, **c** PSP, **d** Pick’s, and **e** control (age-matched, Braak ≤ 2) patients; scale bar = 100 µm. **f** Quantification of CK2-positive tissue normalized to hematoxylin area from control (*n* = 5), AD (*n* = 13), CBD (*n* = 8), PSP (*n* = 7), Pick’s (*n* = 6); *p < 0.05, one-way ANOVA followed by Tukey post hoc test. **g** MMSE score, **h** age of onset (years), and **i** disease duration correlated with CK2-positive area from AD patient samples normalized to hematoxylin area. *p < 0.05, Pearson’s correlation; *n* = 15

Brain tissue from patients neuropathologically diagnosed with AD, CBD, PSP, or Pick’s were used in the immunohistochemical assessment of CK2 in tauopathies (Additional file 4: Table 1). Tauopathy in these patients was characterized as Braak stage V or VI, indicative of extensive spreading of hyperphosphorylated tau from the entorhinal cortex to the visual cortex. These patients were compared to age-matched controls, which were defined as individuals with unremarkable age-related tauopathy indicative of Braak stage I or II. In order to demonstrate that any potential differences in hippocampal CK2 expression were independent of pathological tau load, we first histologically evaluated hyperphosphorylated tau across all four neurodegenerative diseases. Hyperphosphorylated tau was specifically examined at the serine 396/serine 404 phosphorylation site (PHF1) due to its significant relationship to NR2B-mediated processes [64]. Cases from the various tauopathies did not display any significant difference in PHF1 immunoreactivity (Additional file 1: Fig. 1). Notwithstanding comparable amounts of PHF1 across groups, we observed a relative abundance of CK2 only in AD hippocampal tissue (Fig. 1a, f). This indicates that previous reports on the role of CK2 in hyperphosphorylated tau may only be clinically relevant to pathological processes within AD brains.

In addition to neuropathological changes displayed in AD patient brains, clinical changes such as cognitive impairment are well characterized among AD patients. AD patient scores on the Mini-Mental State Exam (MMSE) are associated with both tau burden and NR2B-associated synaptic alterations [8, 9, 18, 43, 52, 54, 74]. Accordingly, we investigated the relationship between CK2 and cognitive function. Hippocampal CK2 expression negatively correlated with AD patient MMSE scores, suggesting that a pathological increase in CK2 may impair hippocampal-dependent cognitive function (Fig. 1g). Clinical studies have also previously linked the onset of cognitive symptoms to disease severity [27, 31, 53]. Therefore, we assessed hippocampal CK2 in the context of disease onset and duration in AD patients. No significant relationship was found between hippocampal CK2 and age of onset or disease duration (Fig. 1i, h). Taken together, these results suggest that AD patient brains may also uniquely exhibit changes in CK2’s downstream effector NR2B compared to other tauopathies.

### AD-tau pathology uniquely correlates with hippocampal NR2B

**Fig. 2:**
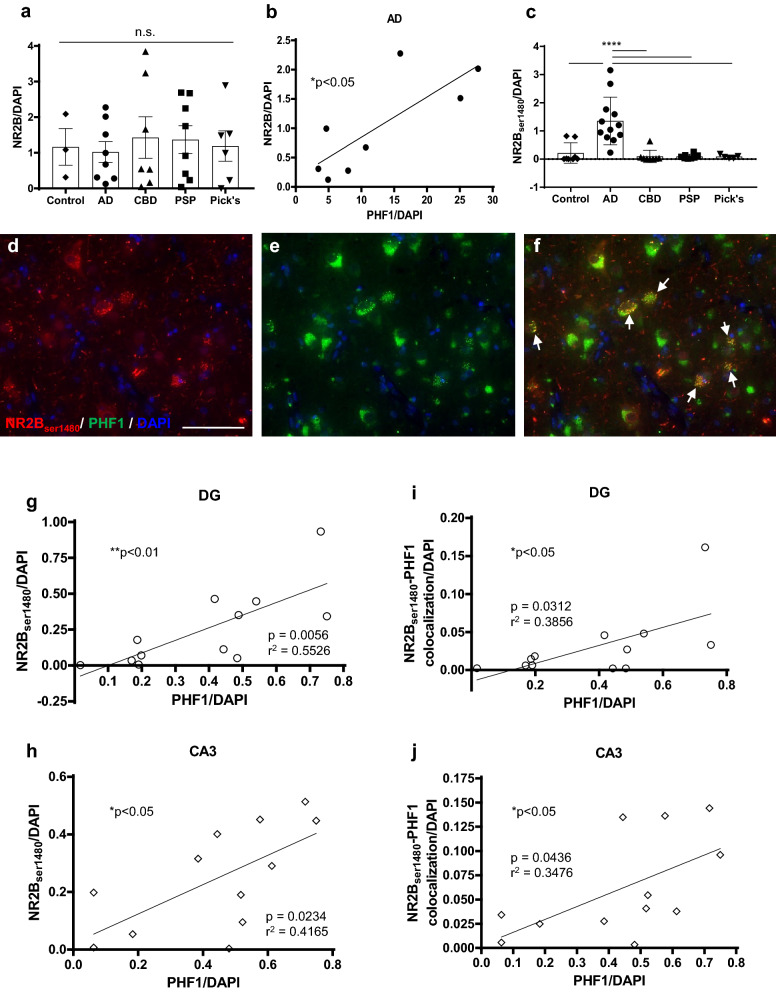
NR2B selectively translocates and correlates with AD-tau. Hippocampal tissue samples from tauopathy patients and age-matched controls were stained with NR2B and counterstained with DAPI. **a** Quantification of NR2B-positive tissue normalized to DAPI from control (age-matched, Braak ≤ 2) (*n* = 3), AD (*n* = 8), CBD (*n* = 6), PSP (*n* = 8), Pick’s (*n* = 6) patient samples. n.s. = nonsignificant; one-way ANOVA followed by Tukey post hoc test. **b** NR2B-positive tissue correlated with PHF1, normalized to DAPI from AD (*n* = 8) hippocampal samples. *p < 0.05, Pearson’s correlation. **c** Quantification of NR2B_ser1480_-positive tissue normalized to DAPI from control (*n* = 8), AD (*n* = 12), CBD (* n* = 8), PSP (*n* = 8), and Pick’s (*n* = 5) patient samples. ****p < 0.0001, one-way ANOVA followed by Tukey post hoc test. **d–f** IHC of NR2B_ser1480_ and PHF1 in AD hippocampus; scale bar = 50 µm; white arrows indicate colocalization. NR2B_ser1480_-positive tissue correlated with PHF1, normalized to DAPI from AD patient tissue in **g** DG and **h** CA3. NR2B_ser1480_ and PHF1-positive tissue correlated with PHF1, normalized to DAPI from AD patient tissue in **i** DG and **j** CA3. *p < 0.05, **p < 0.01, Pearson’s correlation; *n* = 12

Previous studies have investigated the functional role of extrasynaptic and synaptic NR2B in AD, but potential pathological changes in NR2B expression have not yet been evaluated. Further, NR2B expression has not been characterized in other tauopathies despite the presence of synaptic dysfunction in CBD, PSP, and Pick’s [4, 35, 51]. In order to address these unanswered questions, we next investigated relative changes in NR2B expression across AD, CBD, PSP, and Pick’s patient brains. Quantification of hippocampal NR2B demonstrated equal expression of the receptor subunit across all tauopathies as well as age-matched controls (Fig. 2a). We correlated hippocampal NR2B and PHF1 across the above mentioned diseases in order to examine prospective positive or negative relationships between the two proteins. The receptor subunit expression only correlated with hyperphosphorylated tau within AD patient hippocampal samples notwithstanding the absence of significant differences in NR2B immunoreactivity among all individuals (Fig. 2b). No such significant trend was observed in CBD, PSP, or Pick’s patient brains, indicating that NR2B expression exclusively pertains to pathological tau among AD patient brains (additional file 1: Fig. 1).

### AD patient brains uniquely display increased CK2-mediated translocated NR2B that regionally correlates with PHF

In order to assess CK2 specific changes in NR2B phosphorylation across patients we immunohistochemically evaluated hippocampal NR2B_ser1480_ expression. AD patient brains were the only hippocampal samples that demonstrated a significant increase in NR2B_ser1480_, which was consistent with our disease specific observations in aberrant CK2 expression (Fig. 2c). Accordingly, we proceeded to immunohistochemically assess NR2B_ser1480_ expression in the DG, CA3, and CA1 regions of AD patient brains in order to further characterize CK2-mediated translocated NR2B in hippocampal subregions. NR2B_ser1480_ immunoreactivity was observed as diffuse and punctate staining, which colocalized with PHF1 staining (Fig. 2d-f). Quantification did not reveal any regional differences in NR2B_ser1480_ immunoreactivity (Additional file 3: Fig. 3c). Similarly, NR2B_ser1480_ and PHF1 homogenously colocalized throughout the examined hippocampal regions (Additional file 3: Fig. 3e). We next examined potential correlations between NR2B_ser1480_ and hyperphosphorylated tau in each region of the AD cases. The results demonstrate that PHF1 correlates with total NR2B_ser1480_ expression only within the DG and CA3 region of these AD brains (Fig. 2g, h). We also observed a positive correlation between NR2B_ser1480_-PHF1 colocalization and PHF1, which was similarly restricted to the DG and CA3 (Fig. 2i, j). The data indicate that NR2B_ser1480_ is preferentially increased in AD patient hippocampal samples compared to other tauopathies. Moreover, CK2-mediated phosphorylation and concomitant translocation of NR2B is equally expressed throughout the hippocampus in the AD brain. The data also indicate that NR2B_ser1480_ colocalizes with PHF1, which is also observed homogenously across hippocampal regions in AD brains. Despite the lack of regional specificity in hippocampal NR2B_ser1480_ expression, the results demonstrate that NR2B_ser1480_ as well as its colocalization with PHF1 only correlate with pathological tau in the DG and CA3 of AD brains. Taken together, this suggests that increases in the CK2-mediated translocation of synaptic to extrasynaptic NR2B may play a significant role in the formation of pathological AD-tau as well as with the colocalization of NR2B_ser1480_ and PHF1. However, these positive correlations are exclusively observed in the hippocampal DG and CA3 regions of AD brains, suggesting that these significant relationships are critically dependent on the mossy fiber pathway as well as the presence of AD-tau pathology.

### Enzymatically active CK2 colocalizes and correlates with PHF in DG and CA3

**Fig. 3:**
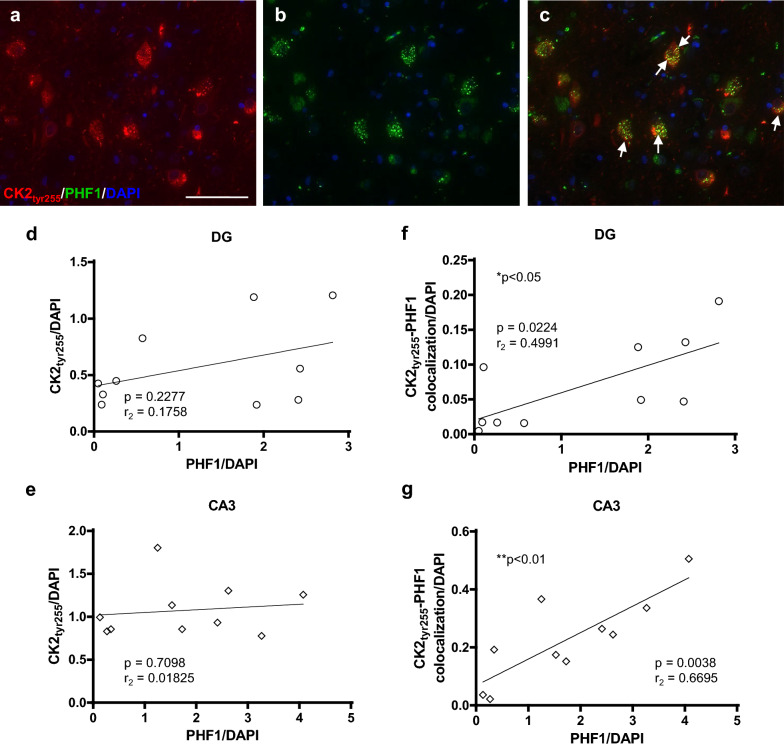
CK2_tyr255_ colocalizes and correlates with AD-tau in DG and CA3 Hippocampal tissue samples from AD patients were stained with CK2_tyr255_ and PHF1 and counterstained with DAPI. **a-c** IHC of CK2_tyr255_ and PHF1 in AD hippocampus; scale bar = 50 µm; white arrows indicate colocalization. CK2_tyr255_-positive tissue correlated with PHF1, normalized to DAPI from AD patient tissue in **d** DG and **e** CA3. CK2_tyr255_ and PHF1-positive tissue correlated with PHF1, normalized to DAPI from AD patient tissue in **f** DG and **g** CA3. *p < 0.05, **p < 0.01, Pearson’s correlation; *n* = 10

Given the disease specific alterations in CK2, we further investigated enzymatically active CK2 expression in AD patient brains. Specifically, we assessed the expression of hippocampal CK2_tyr255_, which is responsible for the serine 1480 phosphorylation and concomitant translocation of NR2B. Immunohistochemical analysis exhibited CK2_tyr255_ punctate immunoreactivity that colocalized with PHF1 in AD hippocampal samples (Fig. 3a–c). PHF1 immunoreactivity remained comparable across the DG, CA3, and CA1 in AD patient brains (Additional file 3: Fig. 3a). Regional expression of CK2_tyr255_, nor its colocalization with PHF1, also did not differ in AD patient hippocampal samples (Additional file 3: Fig. 3b, d). To determine if regional CK2_tyr255_ is related to PHF1 expression in AD patient brains, we correlated the total fluorescent signals of the two phosphorylated proteins (Fig. 3 d, e). The data did not display any significant relationship between CK2_tyr255_ and hyperphosphorylated tau within the DG, CA3, or CA1 brain regions. Notably, positive correlations were revealed upon analyzing the relationship between CK2_tyr255_-PHF1 colocalization. These significant results were regionally dependent, with positive correlations only observed within the DG and CA3 of AD patient brains (Fig. 3 g, h). The significance of colocalization rather than total expression of CK2_tyr255_ suggests that these proteins may be interacting with each other within the hippocampus of AD patient brains. Further, this potential interaction between CK2_tyr255_ and PHF1 may serve as a more accurate predictor of hippocampal AD-tau load than the total amount of enzymatically active CK2. These results also indicate that the relationship between these two phosphorylated proteins is specific to the DG and CA3 in the AD brain. This suggests a distinct role of mossy fiber circuitry for the hippocampal expression of these proteins in AD pathogenesis.

### AD-tau increases CK2 in hippocampal neurons

**Fig. 4:**
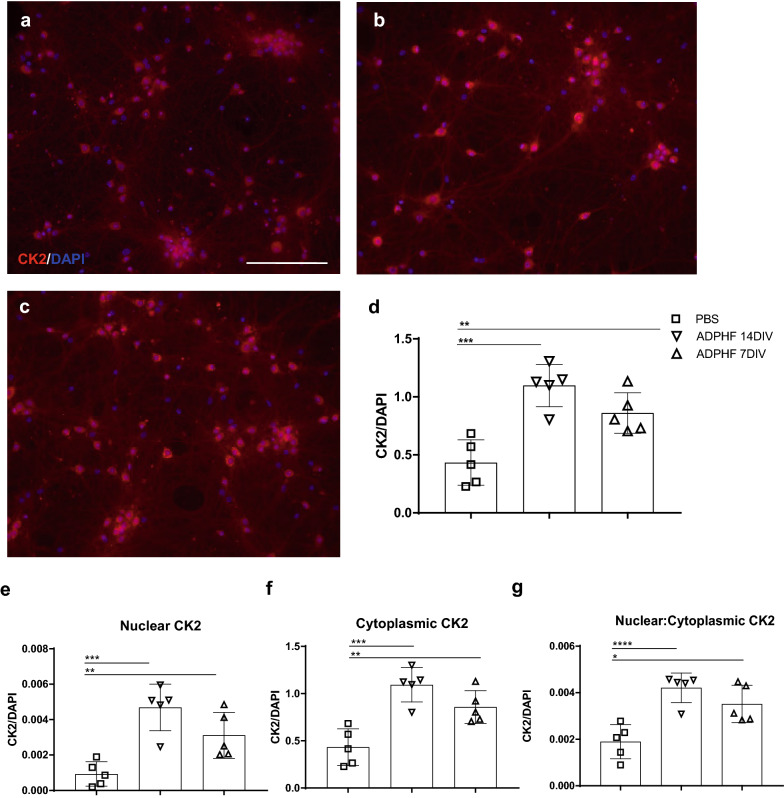
AD-tau increases CK2 in vitro Immunocytochemistry of CK2 in ADPHF treated hippocampal neurons. Neurons were treated with **a** PBS or ADPHFs for **b** 14 or **c** 7 days in vitro (DIV); scale bar = 50 µm. **d** Quantification of CK2-positive area was normalized to DAPI positive area. **p < 0.01, ***p < 0.001, one-way ANOVA followed by Tukey post hoc test. Quantification of **e** nuclear, **f** cytoplasmic, and **g** nuclear:cytoplasmic ratio of CK2 normalized to DAPI. *p < 0.05, **p < 0.01, ***p < 0.001, one-way ANOVA followed by Tukey post hoc test. *n* = 5/group

Aberrant changes in CK2 have been reported in AD patients in addition to AD mouse and cell models. However, such in vivo experiments have not yet investigated the mechanistic role of CK2 in tau pathogenesis independent of amyloid beta (Aβ) plaques, the other primary pathological hallmark of AD. Transgenic AD animal models, such as APP/PS1 and 3xTg mice, are commonly used to study CK2, but such models display Aβ plaques with or without hyperphosphorylated tau [90]. Further, in vitro AD models have demonstrated clinically irrelevant changes in the context of either tau or CK2 overexpression [90]. CK2 expression is yet to be investigated in a translational in vitro model of AD that utilizes the uptake of patient derived AD-tau absent of Aβ. Accordingly, we transduced hippocampal neurons with ADPHFs in order to demonstrate potential clinically relevant changes in CK2 expression. To specifically assess the role of CK2 in tau aggregation, we utilized ADPHFs consisting of pure pathological, insoluble tau without any significant contamination from Aβ (Table 1). We transduced hippocampal neurons with ADPHFs at two time points simulating both an early and late stage of AD. Specifically, neurons transduced at DIV 7 incubated for two weeks (ADPHF 14 DIV) prior to immunocytochemistry and reflected the late stage of AD. Neurons transduced at DIV 14 incubated for one week (ADPHF 7 DIV), reflecting an earlier stage of AD. We hypothesized that ADPHFs would accurately model our previous observations in AD patients. Accordingly, we first hypothesized that ADPHFs would elicit an increase in CK2 immunoreactivity at both time points. We further hypothesized that CK2 expression at these time points would not differ, due to the non-significant correlation between CK2 and disease duration in AD patient hippocampal samples (Fig. 1i). Analysis revealed a significant increase in CK2 immunoreactivity in both the early and late stage groups compared to PBS treated neurons (Fig. 4a–d). As anticipated, the increase in CK2 was comparable in both ADPHF treated groups. These results indicate that this in vitro model replicates the pathological increase in CK2 expression we observed in AD patients.

**Table 1:** Uncontaminated ADPHFs used in study

	Tau	Tau/total protein	Aβ 1–40	Aβ 1–40/total protein	Aβ 1–42	Aβ 1–42/total protein
	mg/ml		mg/ml		mg/ml	
AD case 1	2.1	25.69%	0.000022	0.0002%	0.000102	0.0012%
AD case 2	0.61	11.29%	0.000016	0.0003%	0.000083	0.0015%
AD case 3	0.4	14.29%	0.000032	0.0011%	0.000060	0.0021%
AD case 4	0.53	18.28%	0.000003	0.0001%	0.000061	0.0021%
AD case 5	0.47	27.65	0.000010	0.0005%	0.000095	0.0056%

Newly synthesized individual catalytic alpha subunits of CK2 within the cytoplasm can dynamically transport to and from the nucleus [20]. The holoenzyme comprised of catalytic and regulatory CK2 subunits remains in the cytoplasm to phosphorylate various protein substrates such as NR2B [20]. In order to determine if the observed ADPHF-induced increase in CK2 expression could be attributed to an increase in cytoplasmic catalytic subunits we assessed the nuclear:cytoplasmic ratio. Analysis revealed a significant increase in the nuclear:cytoplasmic ratio of CK2 in both ADPHF treated groups, indicating an increase in nuclear catalytic CK2 subunits (Fig. 4e, g). Notably, results also demonstrated an increase in cytoplasmic CK2 in ADPHF treated groups (Fig. 4f). Thus suggesting that AD-tau may facilitate heightened CK2 activity and CK2-mediated phosphorylation in downstream substrates in vitro.

### CK2 inhibition ameliorates tau burden in hippocampal neurons

**Fig. 5:**
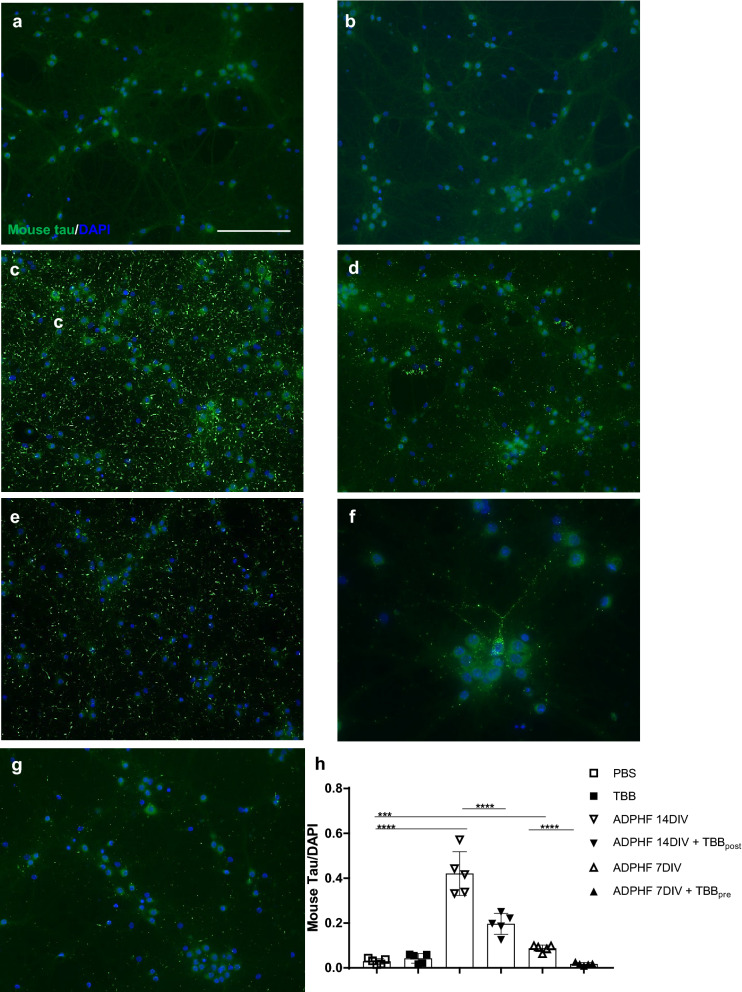
Inhibition of CK2 decreases ADPHF induced tau pathology. Immunocytochemistry of aggregated endogenous mouse tau in A DPHF treated hippocampal neurons. The mouse tau-specific antibody R2295M was used to visualize and quantify tau pathology. Neurons were treated with **a** PBS, **b** TBB, **c ** ADPHF for 14 days in vitro (DIV), **d** ADPHF 14DIV and TBB, **e** ADPHF fo r 7DIV, **f** & **g** ADPHF 7DIV and TBB; scale bar = 50 µm. **h** Quantification of mouse tau (R2295M) normalized to DAPI. ***p < 0.001, * ***p <  0.0001, one- way ANOVA followed by Tukey post hoc test. *n* = 5/group

We have previously demonstrated that the transduction of ADPHFs in hippocampal neurons eventually led to the uptake, aggregation, and propagation of hyperphosphorylated mouse tau [30]. In the present study, this in vitro approach was used to examine any therapeutic effect of CK2 inhibition on pathological AD-tau. We used the same time points for ADPHF transduction to investigate potential beneficial effects of TBB. Hyperphosphorylated tau was characterized as aggregated endogenous mouse tau after ADPHF exposure and concomitant uptake and spread. Similar to previous studies, cells exposed to ADPHF for 14 DIV exhibited a significant increase in pathological tau (Fig. 5c, h) [30, 87]. ADPHF incubation for 7 DIV also yielded a significant increase in aggregated tau at a level that was significantly lower than the other transduced group (Fig. 5e, h). Notably, these aggregates formed a phenotype indicative of cell body AD-tau accumulation (Fig. 5e). This suggests that our model replicates an early stage of AD-tau pathology that shows nascent tau aggregates prior to significant AD-tau propagation. This model was used in conjunction with the CK2 inhibitor TBB in order to investigate the potential therapeutic effect of CK2 inhibition on tau aggregation. TBB was applied to neurons at DIV 14 in order to influence expression levels of CK2 and NR2B indicative of adult developmental levels of both proteins [2, 67]. TBB application was therefore used as both a post-treatment in ADPHF 14 DIV cells as well as a pretreatment in ADPHF 7 DIV cells in order to examine possible therapeutic or prophylactic effects, respectively. We found that TBB had significant effects for treating both groups of AD neurons (Fig. 5e, f, h). Interestingly, some individual neurons in the ADPHF 7 DIV with TBB pretreatment group showed an accumulation of aggregated AD-tau, suggesting that CK2 inhibition may alleviate pathological AD-tau by prohibiting its release and spread (Fig. 5g).

### CK2 inhibition reverses AD-induced mislocalization of NR2B

**Fig. 6:**
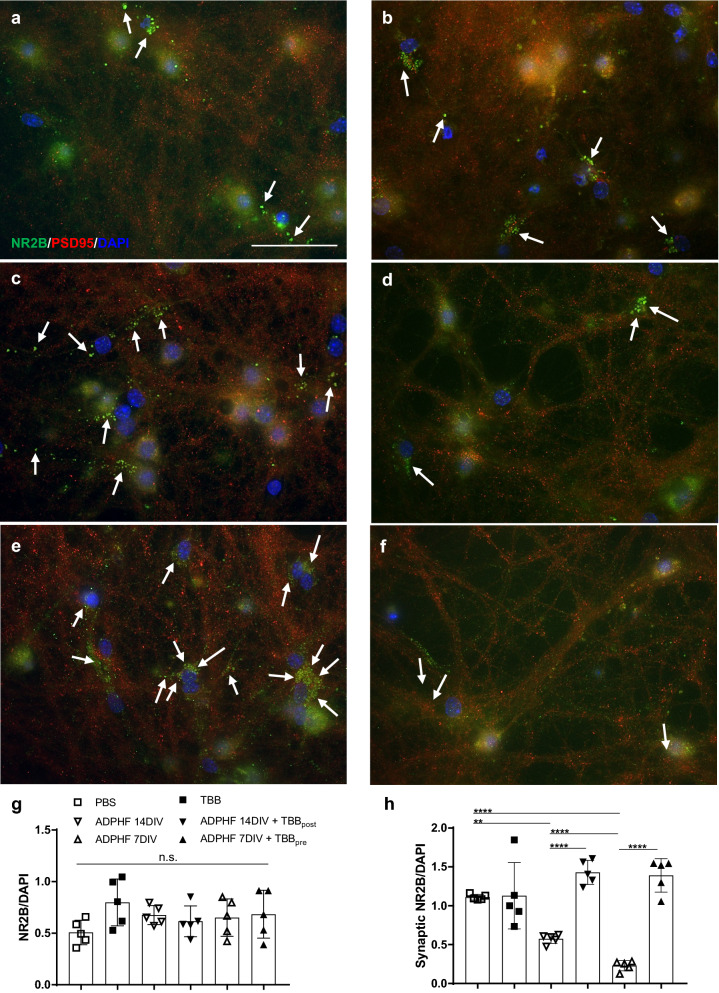
Mislocalization of NR2B in AD patients is replicated by ADPHF transduction in vitro and ameliorated by TBB Immunocytochemistry of synaptic (NR2B-PSD95 colocalization) and extrasynaptic NR2B (white arrows) in ADPHF treated hippocampal neurons. Neurons were treated with **a** PBS, **b** TBB, **c** ADPHF for 14 days in vitro (DIV), **d** ADPHF 14DIV and TBB, **e** ADPHF for 7DIV, **f** ADPHF 7DIV and TBB; scale bar = 50 µm. **g** Quantification of NR2B normalized to DAPI. **h** Quantification of synaptic NR2B normalized to DAPI. **p < 0.01, ****p < 0.0001, one-way ANOVA followed by Tukey post hoc test. *n* = 5/group

Previous reports have demonstrated that activating extrasynaptic NR2B subunits increases hyperphosphorylated tau in cortical neurons [73]. Additionally, past studies have demonstrated that tau knockout mice exhibit dysfunctional extrasynaptic NR2B subunits, but previous studies have not investigated tau-induced changes in NR2B location [55]. In the present study, we used our in vitro experimental approach to assess if ADPHFs promote a shift from synaptic to extrasynaptic NR2B as a potential consequence of the increased CK2 we observed in neurons as well as AD patients. We hypothesized that neurons exposed to ADPHFs would demonstrate an increase in extrasynaptic NR2B as indicated by a loss in synaptic NR2B as well as a loss in cytosolic NR2B. We further hypothesized that ADPHFs would not affect the total amount of NR2B, therefore replicating the physiological amount of NR2B expression observed in AD patient brains earlier. Results demonstrated that ADPHFs did not affect total NR2B expression at either time point of exposure compared to control groups (Fig. 6g). Neurons that were incubated with ADPHFs for 14 days demonstrated a significant loss of synaptic NR2B as indicated by a decrease in NR2B-PSD95 colocalization as well as a significant decrease in NR2B-Golgi and NR2B-ER colocalization (Fig. 6c, h; Additional file 3: Fig. 3). Further, neurons exposed to ADPHFs for only 7 days exhibited an even greater loss of synaptic NR2B compared to control groups (Fig. 6e, h). This indicates that ADPHFs induced a mislocalization of NR2B, without affecting the total expression of the receptor. Differences in these two time points also suggests that pathological changes in synaptic function may be more dysfunctional during early stages of AD. Next, we hypothesized that TBB pre and post-treatment would ameliorate AD related changes in NR2B. Interestingly, using TBB to inhibit 50% of CK2 activity did not alter the location of NR2B in control neurons, but it did ameliorate synaptic changes in both pathological groups. Specifically, TBB normalized the amount of synaptic NR2B as both a pre and post-treatment (Fig. 6e, f, h). This demonstrates that early and late stage changes in synaptic function can be prevented or reversed when treated either before or after the onset of tau aggregation. These results further suggest that treatments at either time points are comparably effective at restoring the physiological ratio of synaptic to extrasynaptic NR2B.

### Therapeutic effect of Memantine requires AD-induced NR2B mislocalization

**Fig. 7:**
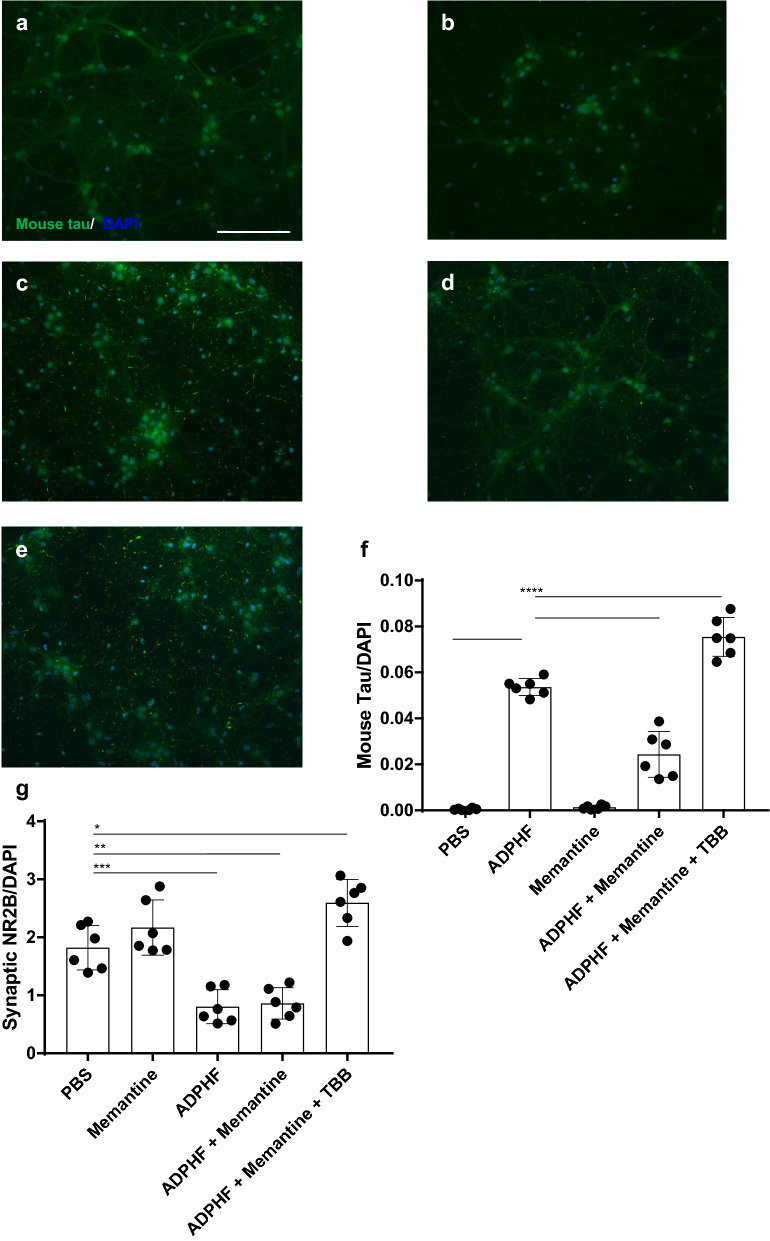
Inhibition of physiological extrasynaptic NR2B exacerbates tau pathology Immunocytochemistry of aggregated endogenous mouse tau in ADPHF treated hippocampal neurons. Neurons were treated with **a** PBS, **b** Memantine, **c** ADPHF for 14 days in vitro (DIV), **d** ADPHF 14DIV and Memantine, **e** ADPHF 14DIV, Memantine, and TBB; scale bar = 50 µm. **f** Quantification of mouse tau normalized to DAPI. **h** Quantification of synaptic NR2B normalized to DAPI. *p < 0.05, **p < 0.01, ^***^p < 0.001, ****p < 0.0001, one-way ANOVA followed by Tukey post hoc test. n = 6/group

Memantine is a noncompetitive NMDA receptor antagonist that selectively inhibits extrasynaptic NR2B receptors and is currently used as an AD therapeutic [21]. Clinical data have reliably demonstrated Memantine’s efficacious ability to improve both MMSE scores and activities of daily living skills in mild to moderate AD patients [16]. Previous in vitro and in vivo studies have demonstrated an ameliorative effect of Memantine on pathological tau [7, 41, 72, 82]. These past approaches do not utilize clinically relevant techniques in generating pathological tau. Further, Memantine-induced inhibition of extrasynaptic NR2B in AD in vitro models would potentially be acting on a pathological increase in extrasynaptic NR2B due to the previously observed AD-tau induced mislocalization of NR2B (Fig. 6c, e, h). In the present study, we utilized Memantine in conjunction with TBB to further assess the role of NR2B location and function in the context of tau pathogenesis. Specifically, this combined pharmacological approach was used to inhibit a physiologically relevant population of extrasynaptic NR2B receptors in the presence of AD-tau. Due to the previously described attenuating effects of Memantine in vitro and our own therapeutic observations of CK2 inhibition on aggregated mouse tau, we hypothesized that further modulation of extrasynaptic NR2B would result in a synergistic effect from the two pharmacological approaches. We utilized ADPHF 14 DIV neurons, which previously demonstrated the greatest increase in aggregated mouse tau, in order to best observe any significant changes in tau accumulation (Fig. 5c, h). As anticipated, Memantine significantly decreased pathological tau without affecting the location of NR2B receptors (Fig. 7d, f, g). In the presence of both Memantine and TBB, ADPHF treated neurons demonstrated a distribution of synaptic and extrasynaptic NR2B receptors comparable to control neurons (Fig. 7g). Interestingly, despite the individual ameliorative effects of Memantine and TBB, this combined treatment significantly increased AD-tau burden compared to non-pharmacologically treated ADPHF neurons (Fig. 7f). These results demonstrate that the inhibition of a physiologically relevant population of extrasynaptic NR2B in the presence of AD-tau facilitates the propagation of tau rather than attenuating the accumulation of the hyperphosphorylated protein. Notably, the reversal of NR2B mislocalization as well as inhibition of excessive extrasynaptic NR2B receptors both alleviated tau. Taken together, this suggests that the balance in synaptic:extrasynaptic NR2B function, rather than expression, modulates tau pathogenesis.

## Discussion

In the present study, we demonstrated that aberrant CK2 expression is a pathological phenomenon unique to AD-tau pathogenesis. Our human data revealed significant changes in CK2 solely in AD patient brains. These results were consistent with previous publications that also demonstrated a significant role of CK2 in AD patient brains as well as animal models [65, 90]. Interestingly, we did not observe similar changes among the other analyzed tauopathies. A potential hypothesis that explains this observation includes other unique features of AD-tau pathology that differentiate the disease from other tauopathies. AD-tau pathology includes both the 3 (3R) and 4 (4R) microtubule-binding repeats, whereas CBD and PSP, and Pick’s disease are primarily affected by the 4R and 3R tau isoforms, respectively [40]. Alternative splicing of MAPT generates tau tangles containing different isoforms of the protein. The inclusion or exclusion of exon 10 yields changes in the production of 3R and/or 4R tau, thereby producing different tauopathies [40]. Accordingly, these diseases may possess isoform-specific pathogenicity. In tauopathy patients, 3R tau correlates with increases in extracellular tau and a shift from 4R to 3R tau corresponds to disease severity [10, 32]. Tau isoforms retain different microtubule binding properties that facilitate unique pathological roles [28]. The expression of 3R tau is associated with increased defects in axonal transport, a neuronal mechanism regulated by CK2 activity [58, 69]. Comparatively, 4R tau has a greater effect on neurodegeneration and cognitive impairments that are associated with NR2B excitoxocity [12, 69]. Therefore, it is possible that proteins such as CK2 and NR2B may play a more significant role in a tauopathy generated by both 3R and 4R tau as opposed to tauopathies composed of a singular predominant tau isoform. Pharmacologically inhibiting extrasynaptic NR2B alleviates excitotoxicity in the CA3 of a mouse model of frontotemporal dementia, another tauopathy consisting of 3R and 4R tau isoforms [12]. In addition, inhibition of extrasynaptic NR2B is neuroprotective in mouse models of chronic traumatic encephalopathy and amyotrophic lateral sclerosis, both of which also represent 3R/4R tauopathies [49, 81]. Further characterization of CK2 and NR2B_ser1480_ is needed in these additional 3R/4R tauopathies.

Our human data also revealed a significant role of the DG and CA3 hippocampal regions in AD patients. Both CK2_tyr255_ and NR2B_ser1480_ had significant interactions with PHF1 within these mossy fiber regions. The mossy fiber pathway is particularly vulnerable in tau pathogenesis and disruption of this hippocampal synaptic circuitry is associated with cognitive impairment in patients and mouse models of AD [13, 14, 84, 85, 89]. Therefore, the observed correlation between these two phosphorylated proteins and tau burden within these distinct hippocampal regions may support the observed correlation between CK2 and MMSE scores. Further, CK2-mediated phosphorylation and translocation of NR2B may contribute to cognitive dysfunction in AD patients. Mossy fiber synaptic dysfunction associated with cognitive impairment is one of the earliest pathological changes in AD [70, 79, 80]. In keeping with these studies, we observed the most significant reduction in synaptic NR2B during the early AD-tau time point, 7 days after ADPHF treatment. Further, this study provides the first evidence that NR2B mislocalizes in any model of AD-tau pathology. The more severe mislocalization of NR2B observed at an earlier time point confirms that synaptic changes in AD are more significant prior to AD-tau aggregation. TBB pretreatment was more efficient at normalizing the location of NR2B in the ADPHF 7 DIV group. This suggests that AD related pathological changes in tau downstream of synaptic dysregulation may be better treated at an early stage of the disease. Further experiments are needed to assess TBB’s therapeutic effect on additional synaptic changes observed in AD.

The early synaptic deficit in conjunction with the absence of changes in total NR2B expression and the pathological increase in CK2 expression further supports the clinical relevance of our patient-derived AD-tau in vitro model. Our study is the first to utilize a clinically relevant in vitro model to demonstrate the ameliorative effect of CK2 inhibition on pathological AD-tau. During the process of cell to cell transmission, AD-tau undergoes aggregation, release, and uptake [24]. Decreases in pathological AD-tau were observed in conjunction with changes in its phenotype after CK2 inhibition. Specifically, hyperphosphorylated AD-tau after TBB pretreatment appeared to accumulate in neurons. This phenotype may reflect a reduction in release, thereby implicating CK2 in this specific step of transmission. CK2 overexpression disrupts fast axonal transport and can induce hyperactivity by decreasing the amount of synaptic NR2B [42, 58]. Further, AD tauopathy models have demonstrated that hyperactivity is capable of driving the release of AD-like tau into the extrasynaptic space [60, 83, 88]. Despite our observations in TBB-mediated changes in NR2B localization being consistent with the mechanism of AD-like tau release, we cannot exclude the possibility that TBB reduced aggregated tau by influencing a more direct connection between NR2B and tau. For example, both hyperphosphorylated tau and internalized NR2B can exist within endosomes, a site known for intracellular toxicity in AD [68, 71]. Further experiments should address the role of CK2 and NR2B in specific stages of AD-tau propagation in order to better understand the progressive nature of these aberrant proteins.

Our in vitro model also facilitated our study on CK2 in the pathological context of tau without compounding variables such as Aβ, contrary to previous studies [90]. Recent clinical studies have identified hyperphosphorylated tau as a better predictor of cognitive decline and brain atrophy comparable to Aβ plaques [5, 38, 54]. These studies demonstrate the significance of elucidating the underlying mechanisms of tau pathogenesis to potentially delay disease progression in AD patients. Our approach enabled the investigation of the relationship between CK2-mediated NR2B translocation in an environment consisting only of aggregated tau, which further elucidated the mechanistic role between extrasynaptic NR2B and pathological tau. Extrasynaptic NR2B is a topic of investigative interest in several neurodegenerative diseases. Specifically, increased extrasynaptic NR2B function due to an increase in extrasynaptic receptors has been proposed as an underlying mechanism for glutamate spillover-induced excitotoxity in proteinopathies such as Huntington’s Disease [50]. According to our findings, it is possible that an increase in extrasynaptic NR2B function may arise in AD patients due to an increase in extrasynaptic receptors, which could facilitate an increase in neurotransmission from glutamate spillover [15, 19, 33, 46, 63]. We demonstrated that inhibition of extrasynaptic NR2B function with Memantine ameliorates tau burden in vitro, but fails to correct AD-induced NR2B mislocalization unlike TBB. Further, combination of both pharmacological treatments exacerbated tau pathology, suggesting that modulating extrasynaptic NR2B function in the presence of balanced synaptic:extrasynaptic receptor expression yields a detrimental imbalance in receptor function. Specifically, the combined treatment’s effect on NR2B function may have generated an aberrant decrease in extrasynaptic function that facilitated AD-tau aggregation.

## Conclusions

In the present study we demonstrate that the previously documented role of aberrant CK2 expression associated with tau pathology is unique to AD-tau pathogenesis among patients neuropathologically diagnosed with various tauopathies. We also provide both patient and in vitro novel evidence of AD-induced changes in NR2B receptor location rather than expression. Our findings support the well-established implications of extrasynaptic NR2B in AD while also illuminating the physiological expression levels observed in the diseased state. We further found that pharmacologically modulating either NR2B extrasynaptic expression or activity yielded a reduction in AD-tau aggregation, whereas the combined pharmacological treatment revealed a deleterious effect on tau burden. Although we observed a decrease in AD-tau aggregation with Memantine treatment alone in vitro, Memantine is not a disease modifying treatment in AD patients [76]. Therefore, we suggest that the balance of NR2B function as well as expression, a state achieved by CK2 inhibition, may be more therapeutically advantageous in clinical studies. The therapeutic effects of CK2 inhibition observed in this study have implications for both the expeditious treatment of sporadic AD and preventative therapy for familial AD. Future work should investigate the mitigating effect of CK2 inhibitors on additional neuropathological characteristics of AD.

## Supplementary Information

**Additional file 1: Fig. 1**. NR2B and PHF1 across tauopathies. Hippocampal tissue samples from tauopathy patients and age-matched controls were stained with NR2B, PHF1, or fluorescently double labeled. NR2B counterstained with hematoxylin in **a** AD, **b** CBD, **c** PSP, **d** Pick’s and **e** control (age-matched, Braak <2) patients; scale bar = 100µm. **f** Quantification of PHF1-positive hippocampal tissue in AD (n = 13), CBD (n = 8), PSP (n = 7), and Pick’s (n = 6) patients. Correlation between NR2B and PHF1 positive area normalized to DAPI positive area in **g** CBD (n = 7), **h** PSP (n = 8) and **i** Pick’s (n = 6) patients. One-way ANOVA followed by Tukey post hoc test; Pearson’s correlation.

**Additional file 2: Fig. 2**. Homogenous subregional hippocampal expression of PHF1, CK2_tyr255_, and NR2B_ser1480_ in AD patients. Hippocampal tissue samples from AD patients stained with PHF1, CK2_tyr255_, NR2B_ser1480_, or fluorescently double labeled with PHF1 and CK2_tyr255_ or NR2B_ser1480_. Quantification of **a** PHF1 positive area (n = 16), **b** CK2_tyr255_ positive area (n = 10), **c** NR2B_ser1480_ positive area (n = 12), **d** CK2_tyr255_ and PHF1 positive area (n = 10), and **e** NR2B_ser1480_ and PHF1 positive area (n = 12) normalized to DAPI positive area from DG, CA3, and CA1 subregions. One-way ANOVA followed by Tukey post hoc test.

**Additional file 3: Fig. 3**. AD-tau decreases cytosolic NR2B. Analysis of immunocytochemistry of NR2B colocalization with Golgi and endoplasmic reticulum organelle markers. Quantification of **a** NR2B and GM130 positive area and **b** NR2B and KDEL positive area normalized to DAPI. *p<0.05, **p<0.01, ****p<0.0001, one-way ANOVA followed by Tukey post hoc test. n = 6/group.

**Additional file 4: Table 1**. Patient Demographics

**Additional file 5: Table 2**. Primary antibodies

## Data Availability

The data sets during and/or analyzed during the current study available from the corresponding from the corresponding author on reasonable request.

## Abbreviations

AD: (Alzheimer’s disease)
ADPHF: (AD patient derived paired helical filaments)
CA3: (Cornu ammonis 3)
CBD: Corticobasal degeneration
CK2: (Casein Kinase 2)
DG: (Dentate gyrus)
LTD: (Long term depression)
NFT: (Neurofibrillary tangles)
PHF: (Paired helical filaments)
PHF1: (Antibody for tau phosphorylated at serine 396/404)
Pick’s: Pick’s disease
PSP: Progressive supranuclear palsy
TBB: (4,5,6,7-Tetrabromo-1H-benzotriazole)
3R: (3 Repeat tau isoform)
4R: (4 Repeat tau isoform)
