# Supplementary figures and images for: Inhibition of CK2 mitigates Alzheimer’s tau pathology by preventing NR2B synaptic mislocalization
Source: Acta Neuropathol Commun. 2022 Mar 4;10:30. doi: 10.1186/s40478-022-01331-w (PMC8895919; doi:10.1186/s40478-022-01331-w)

## Slide 1
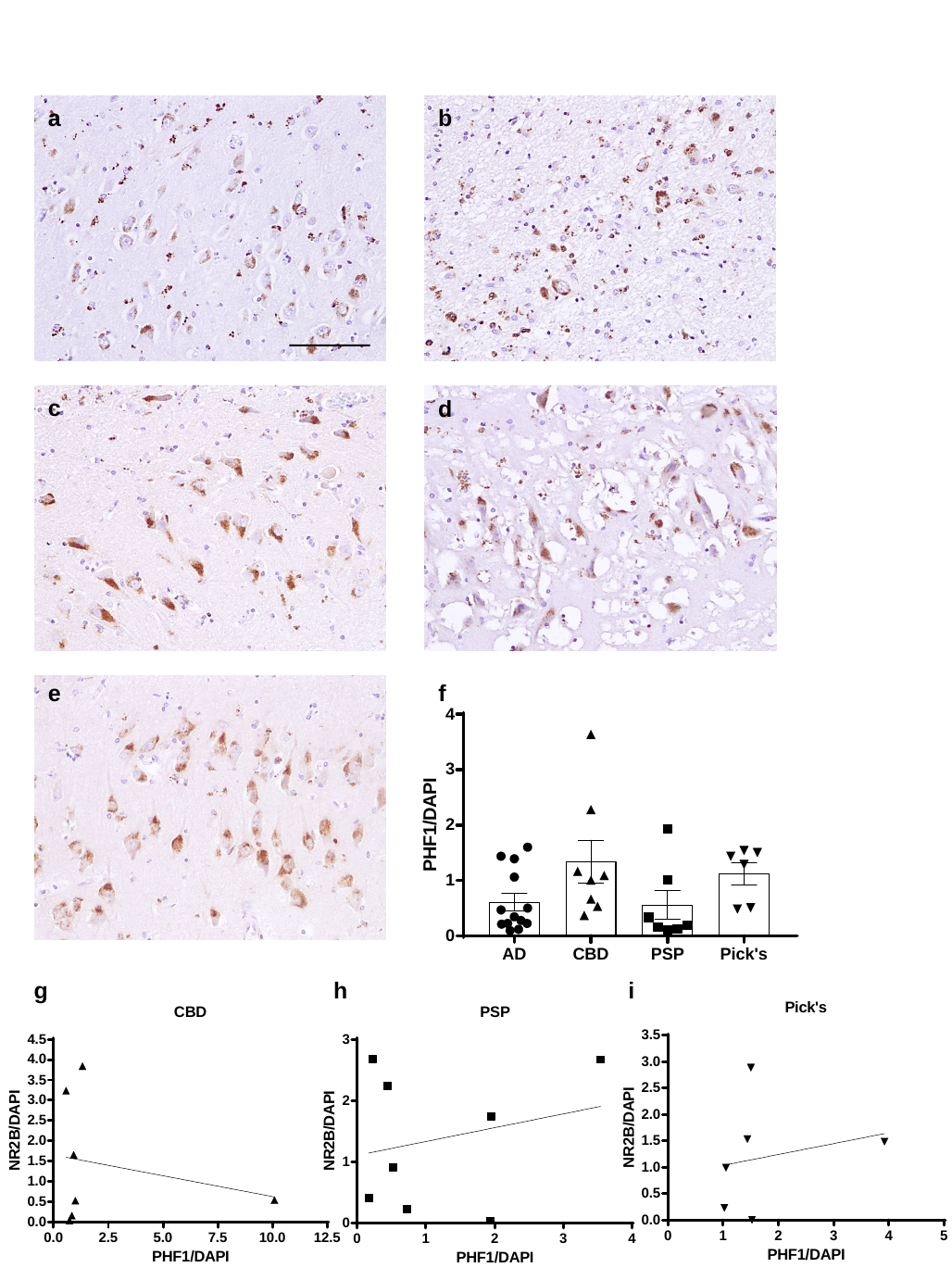

a
b
c
d
e
f
h
g
i

Supplement: Supplementary file 1 — Additional file 1: Fig. 1. NR2B and PHF1 across tauopathies. Hippocampal tissue samples from tauopathy patients and age-matched controls were stained with NR2B, PHF1, or fluorescently double labeled. NR2B counterstained with hematoxylin in a AD, b CBD, c PSP, d Pick’s and e control (age-matched, Braak <2) patients; scale bar = 100µm. f Quantification of PHF1-positive hippocampal tissue in AD (n = 13), CBD (n = 8), PSP (n = 7), and Pick’s (n = 6) patients. Correlation between NR2B and PHF1 positive area normalized to DAPI positive area in g CBD (n = 7), h PSP (n = 8) and i Pick’s (n = 6) patients. One-way ANOVA followed by Tukey post hoc test; Pearson’s correlation. [file 40478_2022_1331_MOESM1_ESM.pptx]

## Slide 1
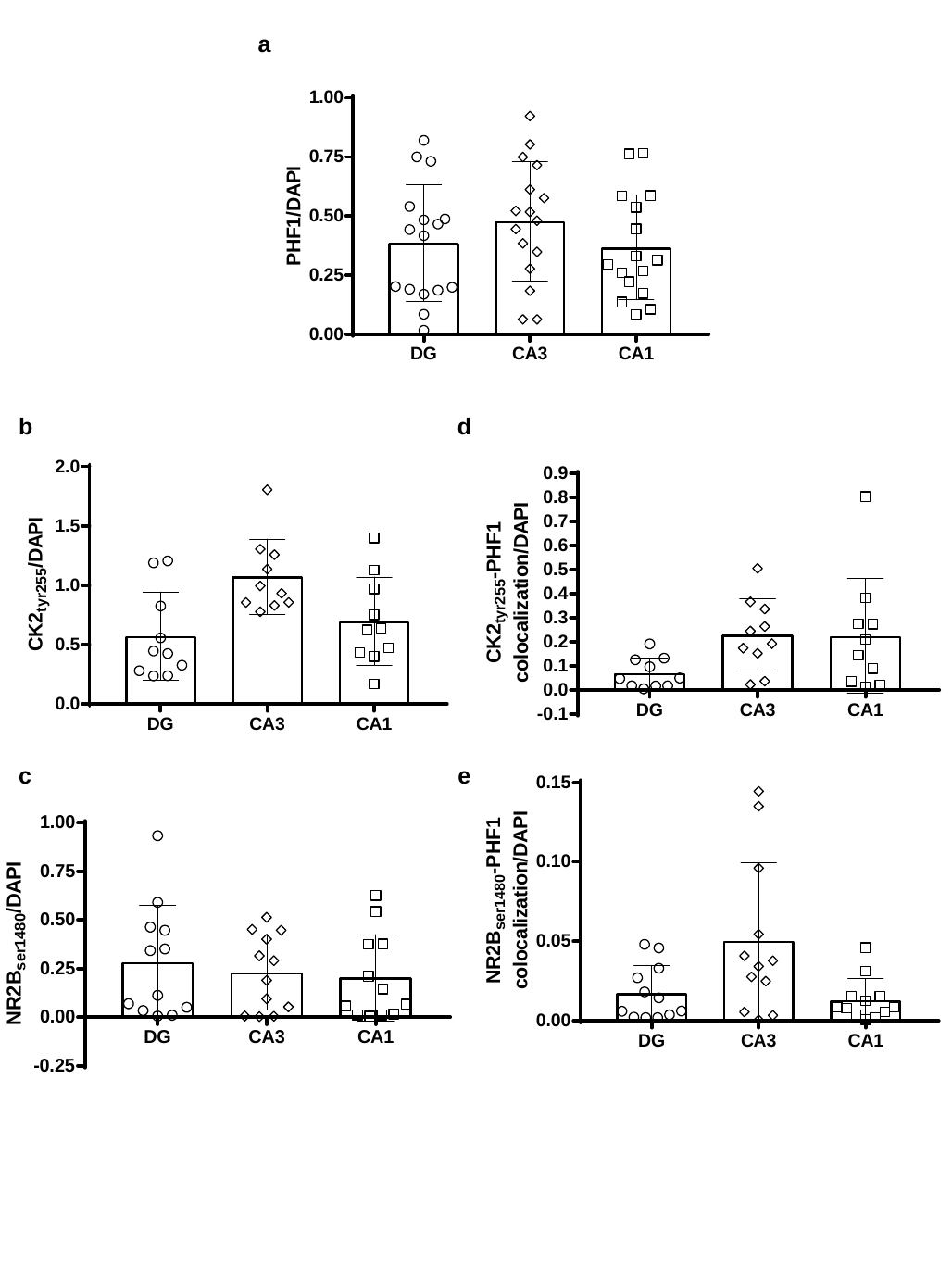

a
d
b
e
c

Supplement: Supplementary file 2 — Additional file 2: Fig. 2. Homogenous subregional hippocampal expression of PHF1, CK2tyr255, and NR2Bser1480 in AD patients. Hippocampal tissue samples from AD patients stained with PHF1, CK2tyr255, NR2Bser1480, or fluorescently double labeled with PHF1 and CK2tyr255 or NR2Bser1480. Quantification of a PHF1 positive area (n = 16), b CK2tyr255 positive area (n = 10), c NR2Bser1480 positive area (n = 12), d CK2tyr255 and PHF1 positive area (n = 10), and e NR2Bser1480 and PHF1 positive area (n = 12) normalized to DAPI positive area from DG, CA3, and CA1 subregions. One-way ANOVA followed by Tukey post hoc test. [file 40478_2022_1331_MOESM2_ESM.pptx]

## Slide 1
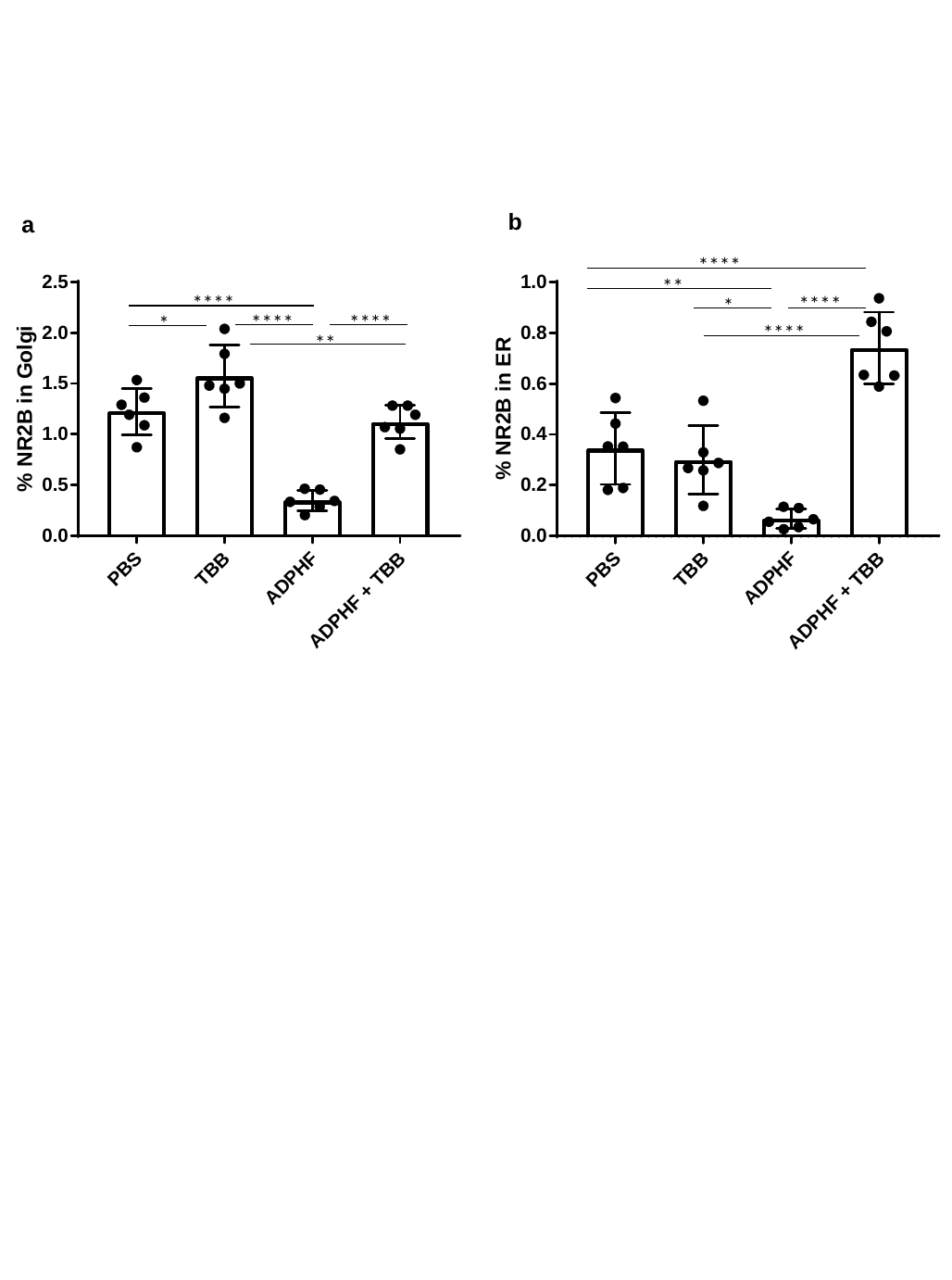

b
a
****
**
****
****
*
****
****
*
****
**

Supplement: Supplementary file 3 — Additional file 3: Fig. 3. AD-tau decreases cytosolic NR2B. Analysis of immunocytochemistry of NR2B colocalization with Golgi and endoplasmic reticulum organelle markers. Quantification of a NR2B and GM130 positive area and b NR2B and KDEL positive area normalized to DAPI. *p<0.05, **p<0.01, ****p<0.0001, one-way ANOVA followed by Tukey post hoc test. n = 6/group. [file 40478_2022_1331_MOESM3_ESM.pptx]
