# Supplementary material for: Inhibition of CK2 mitigates Alzheimer’s tau pathology by preventing NR2B synaptic mislocalization
Source: Acta Neuropathol Commun. 2022 Mar 4;10:30. doi: 10.1186/s40478-022-01331-w (PMC8895919; doi:10.1186/s40478-022-01331-w)
Supplement: Supplementary file 4 — Additional file 4: Table 1. Patient Demographics [file 40478_2022_1331_MOESM4_ESM.pptx]

## Slide 1
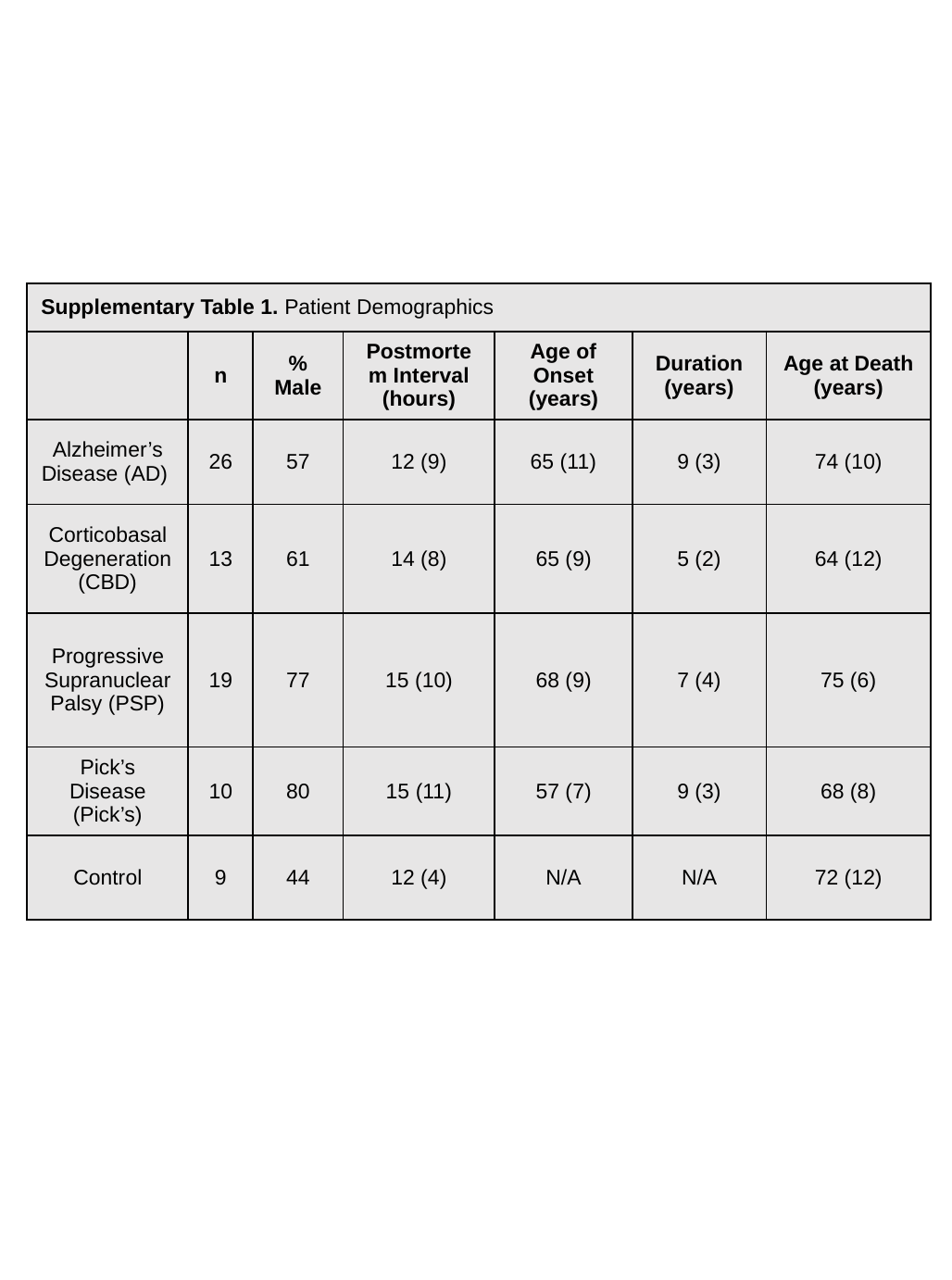

| Supplementary Table 1. Patient Demographics | | | | | | |
| --- | --- | --- | --- | --- | --- | --- |
| | n | % Male | Postmortem Interval (hours) | Age of Onset (years) | Duration (years) | Age at Death (years) |
| Alzheimer’s Disease (AD) | 26 | 57 | 12 (9) | 65 (11) | 9 (3) | 74 (10) |
| Corticobasal Degeneration (CBD) | 13 | 61 | 14 (8) | 65 (9) | 5 (2) | 64 (12) |
| Progressive Supranuclear Palsy (PSP) | 19 | 77 | 15 (10) | 68 (9) | 7 (4) | 75 (6) |
| Pick’s Disease (Pick’s) | 10 | 80 | 15 (11) | 57 (7) | 9 (3) | 68 (8) |
| Control | 9 | 44 | 12 (4) | N/A | N/A | 72 (12) |
