# Supplementary material for: Inhibition of CK2 mitigates Alzheimer’s tau pathology by preventing NR2B synaptic mislocalization
Source: Acta Neuropathol Commun. 2022 Mar 4;10:30. doi: 10.1186/s40478-022-01331-w (PMC8895919; doi:10.1186/s40478-022-01331-w)
Supplement: Supplementary file 5 — Additional file 5: Table 2. Primary antibodies [file 40478_2022_1331_MOESM5_ESM.pptx]

## Slide 1
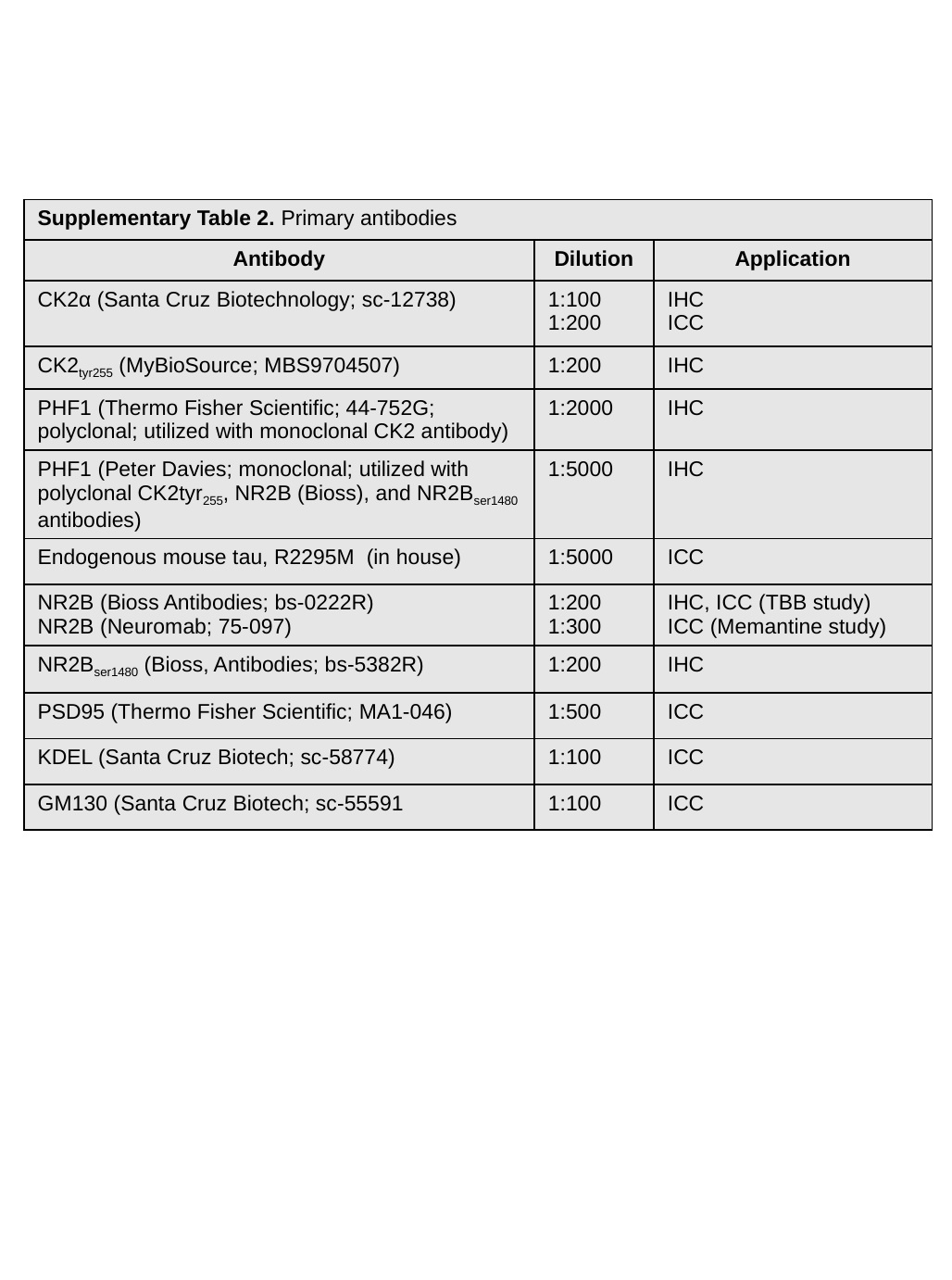

| Supplementary Table 2. Primary antibodies | | |
| --- | --- | --- |
| Antibody | Dilution | Application |
| CK2α (Santa Cruz Biotechnology; sc-12738) | 1:100 1:200 | IHC ICC |
| CK2tyr255 (MyBioSource; MBS9704507) | 1:200 | IHC |
| PHF1 (Thermo Fisher Scientific; 44-752G; polyclonal; utilized with monoclonal CK2 antibody) | 1:2000 | IHC |
| PHF1 (Peter Davies; monoclonal; utilized with polyclonal CK2tyr255, NR2B (Bioss), and NR2Bser1480 antibodies) | 1:5000 | IHC |
| Endogenous mouse tau, R2295M (in house) | 1:5000 | ICC |
| NR2B (Bioss Antibodies; bs-0222R) NR2B (Neuromab; 75-097) | 1:200 1:300 | IHC, ICC (TBB study) ICC (Memantine study) |
| NR2Bser1480 (Bioss, Antibodies; bs-5382R) | 1:200 | IHC |
| PSD95 (Thermo Fisher Scientific; MA1-046) | 1:500 | ICC |
| KDEL (Santa Cruz Biotech; sc-58774) | 1:100 | ICC |
| GM130 (Santa Cruz Biotech; sc-55591 | 1:100 | ICC |
